# High Anti-Viral Protection without Immune Upregulation after Interspecies *Wolbachia* Transfer

**DOI:** 10.1371/journal.pone.0099025

**Published:** 2014-06-09

**Authors:** Ewa Chrostek, Marta S. P. Marialva, Ryuichi Yamada, Scott L. O'Neill, Luis Teixeira

**Affiliations:** 1 Instituto Gulbenkian de Ciência, Oeiras, Portugal; 2 Department of Metabolism and Aging, The Scripps Research Institute, Jupiter, Florida, United States of America; 3 School of Biological Sciences, University of Queensland, St. Lucia, Australia; 4 School of Biological Sciences, Monash University, Clayton, Australia; International Atomic Energy Agency, Austria

## Abstract

*Wolbachia*, endosymbionts that reside naturally in up to 40–70% of all insect species, are some of the most prevalent intracellular bacteria. Both *Wolbachia w*Au, naturally associated with *Drosophila simulans*, and *w*Mel, native to *Drosophila melanogaster*, have been previously described to protect their hosts against viral infections. *w*Mel transferred to *D. simulans* was also shown to have a strong antiviral effect. Here we directly compare one of the most protective *w*Mel variants and *w*Au in *D. melanogaster* in the same host genetic background. We conclude that *w*Au protects better against viral infections, it grows exponentially and significantly shortens the lifespan of *D. melanogaster*. However, there is no difference between *w*Mel and *w*Au in the expression of selected antimicrobial peptides. Therefore, neither the difference in anti-viral effect nor the life-shortening could be attributed to the immune stimulation by exogenous *Wolbachia*. Overall, we prove that stable transinfection with a highly protective *Wolbachia* is not necessarily associated with general immune activation.

## Introduction

*Wolbachia*, intracellular bacteria inhabiting up to 40–70% of known insect species [1], [2], have been initially described as powerful manipulators of arthropods reproduction [3]. *Wolbachia* are maternally transmitted and, in some hosts, provide infected females with a relative fitness advantage by cytoplasmic incompatibility, male killing or other forms of reproductive manipulation. Recently, *Wolbachia* have been attracting widespread attention due to their ability to protect their hosts against viral infections. This phenomenon has been initially reported in *Drosophila melanogaster* carrying its natural *w*Mel *Wolbachia* strain [4], [5]. Interestingly, antiviral protection was the first phenotype of *Wolbachia* discovered in *D. melanogaster* that could explain high prevalence of the symbiont in natural populations of fruit flies [6]–[13].

The ubiquity of *D. melanogaster* in research has placed *w*Mel *Wolbachia* strain among the most extensively studied insect symbionts. Based on the molecular markers it has been shown that *w*Mel strain consists of five polymorphic variants, namely: *w*Mel, *w*Mel2, *w*Mel3, *w*MelCS and *w*MelCS2 [14]. Our previous work [15] has placed these variants in the context of a recent *w*Mel phylogenetic analysis [16] and shown that they cluster into two monophyletic groups: *w*Mel-like and *w*MelCS-like. The *w*MelCS-like variants reach higher densities in the host and provide more antiviral protection than the *w*Mel-like variants. Moreover, some *w*MelCS-like variants shorten the lifespan of their hosts [15], including the extreme example of the pathogenic *w*MelPop [17].

*w*Au is a *Wolbachia* native to *D. simulans* that used to be present at low frequencies in Australia and does not induce cytoplasmic incompatibility [18], [19]. Based on the analyses employing molecular markers different authors concluded that *w*Mel of *D. melanogaster* and *w*Au of *D. simulans* are closely related and both belong to the *Wolbachia* supergroup A [20]–[24].

*w*Au and one of the most protective *Wolbachia w*Mel variants - *w*MelCS_b, the two strains used in this study, have been previously described as protective against *Drosophila* C virus (DCV) and flock house virus (FHV) [4], [5], [25], [26]. Moreover, *w*Mel has been previously transferred from *D. melanogaster* to *D. simulans* [27] and protection in this new *Drosophila*-host association was similar to the protection provided by *w*Au in its natural host [25]. However, different *Wolbachia* lines were studied in different *D. simulans* genetic backgrounds, preventing direct comparison of the protective abilities of *w*Au and *w*Mel.

This study compares the antiviral protection and other phenotypes provided by *w*MelCS_b and *w*Au in genetically identical *D. melanogaster* hosts. In mosquitoes recently transinfected with *Wolbachia* the antiviral effect is frequently associated with activation of the host immune system [29]–[35], while in natural co-evolved *D. melanogaster – Wolbachia* associations antiviral protection is strong but expression of immune genes remains unchanged [31], [36]–[38]. Therefore we also evaluated general activation of the fly immune system by *w*MelCS_b and *w*Au transinfected to *D. melanogaster*.

## Results and Discussion

### *w*Au provides stronger antiviral protection than *w*MelCS_b in *D. melanogaster*

It was previously shown that *w*Au provides strong protection against viruses in its native *D. simulans* host [25]. We have discovered that among *Wolbachia* endosymbionts of *Drosophila melanogaster w*MelCS_b is one of the most potent in viral interference [15]. In order to directly compare these two strains in *Drosophila melanogaster*, we used *Wolbachia*-infected lines in a genetically identical DrosDel *w* ^1118^ isogenic background [39]. *w*MelCS_b was naturally associated with this background while *w*Au was introduced from *D. simulans* to *D. melanogaster* [28] and subsequently placed in this background by chromosome replacement using balancers. A *Wolbachia*-free line, designated “*iso*”, was used as a control in all experiments. All flies were virus-free and had homogenized gut microbiota (see [15]).

**Figure 1:**
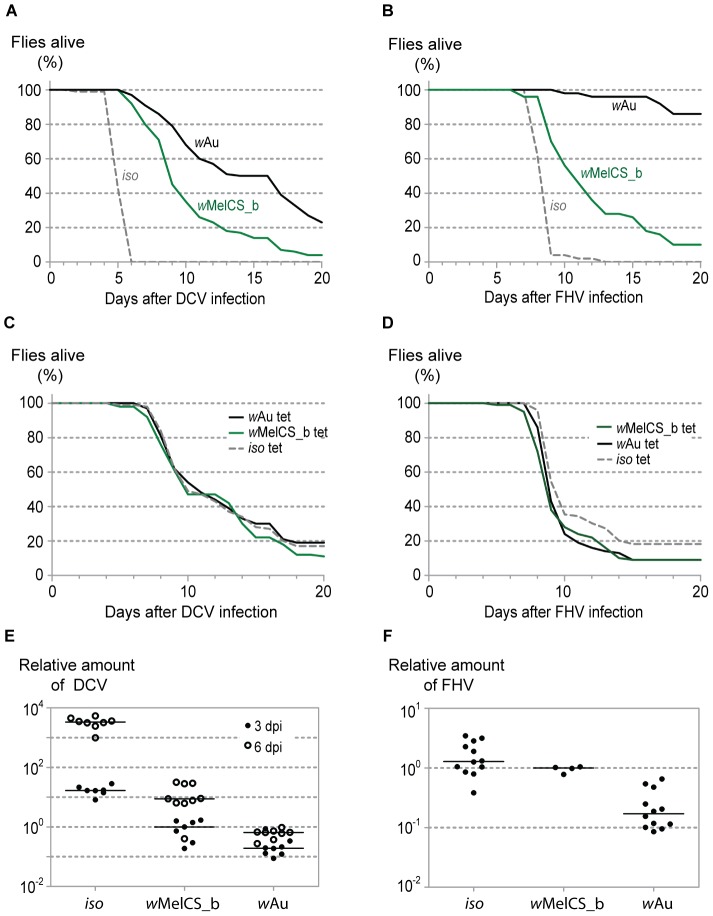
*w*Au provides more antiviral resistance than *w*MelCS_b in *D. melanogaster*. (A) One hundred *Wolbachia*-free *iso*, *w*Au and *w*MelCS_b infected male flies were pricked with DCV (10^9^ TCID_50_/ml) and survival was followed daily. This experiment was repeated twice and statistical analysis was performed on the data from all 3 repetitions (Figure S1A). (B) Fifty *Wolbachia*-free *iso*, *w*Au and *w*MelCS_b infected male flies were pricked with FHV (10^8^ TCID_50_/ml) and survival was followed daily. This experiment was repeated and statistical analysis was performed on the data from both repetitions (Figure S1B). (C) One hundred males from *w*Au, *w*MelCS_b and *iso* tetracycline-treated lines were pricked with DCV (10^7^ TCID_50_/ml) and survival was followed daily. This experiment was repeated and statistical analysis was performed on the data from both repetitions (Figure S1C). (D) One hundred males from *w*Au, *w*MelCS_b and *iso* tetracycline-treated stocks were pricked with FHV (10^8^ TCID_50_/ml) and survival was followed daily. For data analysis see Figure S1D. (E) *Wolbachia*-free *iso*, *w*Au and *w*MelCS_b carrying male flies were pricked with DCV (10^9^ TCID_50_/ml) and collected 3 and 6 days later for RNA extraction and RT-qPCR. Relative amounts of DCV were calculated using host Rpl32 mRNA as a reference and presented values are relative to median of *w*MelCS_b samples 3 dpi. Each point represents a replicate (ten males per replicate, 8 replicates per *Drosophila* line per time point), and lines are medians of the replicates. DCV loads are significantly different between the lines with *w*Au and *w*MelCS_b both 3 dpi (pairwise Wilcoxon rank sum test, *p* = 0.03) and 6 dpi (pairwise Wilcoxon rank sum test, *p*<0.001). (F) *Wolbachia*-free *iso*, *w*Au and *w*MelCS_b carrying male flies were pricked with FHV (10^9^ TCID_50_/ml) and collected 3 days post infection for RNA extraction and RT-qPCR. Relative amount of virus were calculated using host Rpl32 mRNA as a reference and presented values are relative to median of *w*MelCS_b samples. Each point represents a replicate (ten males per replicate), and lines are medians of the replicates. FHV loads are significantly lower in flies with *w*Au comparing to flies with *w*MelCS_b (pairwise Wilcoxon rank sum test, *p* = 0.003).

To compare antiviral properties of *w*MelCS_b and *w*Au, we challenged the flies carrying the respective *Wolbachia* strains and *iso* controls with two viruses: DCV (Figure 1A), a natural pathogen of *Drosophila*, and FHV (Figure 1B), initially isolated from a coleopteran host, but now widely used in studies on dipteran immune response. We observed that *w*Au significantly prolongs the survival of the infected flies in comparison with both *iso* and *w*MelCS_b carrying flies (Figure 1A, S1A, 1B and S1B; Tukey's test on the mixed effects Cox model fit, *w*Au versus both, *w*MelCS_b and *iso*, for DCV: *p*<0.001; for FHV: *p*<0.001). This effect is almost completely abolished in tetracycline-treated flies derived from *Wolbachia*-positive stocks (Figures 1C, S1C, 1D and S1D; DCV infected *w*Au tet *vs iso* tet, *p* = 0.0774 and *w*Au tet *vs w*MelCS_b tet, *p* = 0.0161; FHV infected *w*Au tet *vs iso* tet, *p* = 0.1147 and *w*Au tet *vs w*MelCS_b tet, *p* = 0.8881). The difference between *w*Au tet and *w*MelCS_b tet is very small in the case of DCV infection (compare Figures S1A and S1C).

The strong inhibition of virus-induced mortality in *w*Au carrying flies could be either due to the direct reduction of pathogen load (resistance) or due to neutralization of negative impact of the pathogen on the fly's health without direct influence on the virus titres (tolerance or resilience). To distinguish between these two possibilities we tested the levels of each virus in whole flies either 3 and 6 days post infection (dpi) for DCV or 3 dpi for FHV (Figures 1E and 1F). Consistent with previous reports both *Wolbachia* strains reduce the DCV load. However, this effect is much stronger for *w*Au, which is approximately 4.5 times more efficient 3 dpi (pairwise Wilcoxon rank sum test, *p* = 0.03) and over 13 times more efficient 6 dpi (pairwise Wilcoxon rank sum test, *p*<0.001) in reducing the DCV titres than *w*MelCS_b. Flies carrying *w*Au have also 5.8 times less FHV 3 days after infection in comparison with *w*MelCS_b (pairwise Wilcoxon rank sum test, *p* = 0.003). All these data allow us to conclude that *w*Au protects better against viral infections than one of the most protective *w*Mel variants and this can be, at least partially, explained by the reduction of the viral titres.

### *w*Au reduces the lifespan of *D. melanogaster* and grows exponentially

**Figure 2:**
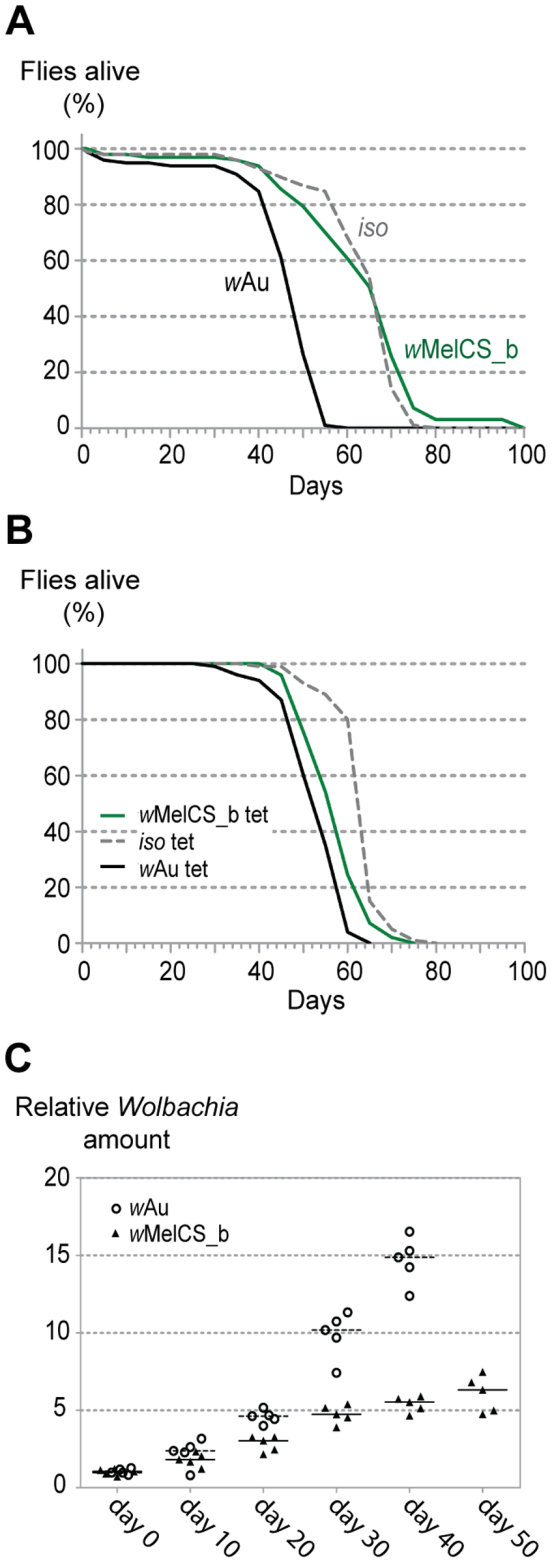
*w*Au shortens the lifespan of the flies and grows exponentially within the hosts. (A) The survival of one hundred *Wolbachia*-free *iso*, *w*Au and *w*MelCS_b carrying male flies was checked every five days. The experiment was repeated once with comparable results and analysis was performed on both repetitions (Figure S1E). (B) The survival of one hundred males derived from *iso*, *w*Au and *w*MelCS_b tetracycline-treated stocks was checked every five days. The experiment was repeated once with comparable results and analysis was performed on both repetitions (Figure S1F). (C) qPCR on DNA isolated from males of *w*MelCS_b and *w*Au lines, collected every 10 days. Day 0 corresponds to 3–6 days-old flies, after day 40 the *w*Au carrying flies were not collected due to the high mortality. Each point represents a sample (each sample consisted of ten males), and lines are medians of the samples. Relative amount of *Wolbachia* genomic DNA was calculated using host Rpl32 as a reference gene and all values are relative to median of samples of *w*MelCS_b at day 0.

We have previously reported the cost of antiviral protection in terms of reduced longevity for some *w*MelCS-like *Wolbachia* variants [15]. Here we have also tested the longevity of the *Wolbachia* infected flies in the absence of viral challenge (Figure 2A). We observed that *w*Au shortens the lifespan of flies by 20 days (31% difference in median time to death) in comparison with *w*MelCS_b (Figure 2A, S1E; Tukey's test on the mixed effects Cox model fit, *w*Au versus *w*MelCS_b and *iso*, *p*<0.001) demonstrating that harbouring this protective endosymbiont is associated with a cost in the absence of infection. After elimination of *Wolbachia* from our fly stocks the flies derived from the *w*Au line also live shorter, but there is only a 5 days difference (9% in median time to death) between them and *w*MelCS_b derived flies (Figure 2B). Despite being smaller, this effect is also significant (Figure S1F; Tukey's test on the mixed effects Cox model fit, *w*Au tet versus *w*MelCS_b tet and *iso* tet, *p*<0.001). This difference and the one we observed for the DCV-infected tetracycline treated *w*Au and *w*MelCS_b lines may be due to differences in mitochondria between *w*Au and *w*MelCS_b fly stocks (see [40], [41]) or to a mutation in the nuclear genetic background that could have arisen since the lines were separated. Given these results we cannot completely rule out an interaction between these possible mitochondrial or nuclear variation and *Wolbachia* as the cause of the differential phenotypes seen in the presence of *Wolbachia*.

The association between *Wolbachia* densities and the strength of antiviral-protection is well established. Various experimental approaches, i.e. treatment of *Wolbachia*-infected flies with increasing antibiotic concentrations or examining natural variation in endosymbiont density, have shown that the higher the *Wolbachia* density, the stronger the antiviral protection [15], [25], [26], [42], [43]. In order to assess if *w*Au titres were also higher than *w*MelCS_b titres, we tested the densities of these symbionts throughout their host's lifespan (Figure 2C). We observed that the *Wolbachia* densities at adult emergence are the same for both strains (log-linear model, intercept difference: 0.165027, *p* = 0.352), but *w*Au grows much faster than *w*MelCS_b (slope difference between *w*Au and *w*MelCS_b: 0.046097, *p*<0.001). The exponential growth of the symbiont may be the cause of the life-shortening, either by direct tissue damage or by constituting a significant metabolic burden compromising the insect's health. This is reminiscent of host life-shortening by the exponentially growing *w*MelPop strain [15], [17], [44].

### *w*Au does not stimulate *D. melanogaster* immune system despite recent transfer from *D. simulans*

Immune upregulation has been shown to occur after transfer of *Wolbachia* into a new insect species [29]–[35]. Stimulation of the insect immune system by *Wolbachia* is one of the proposed mechanisms explaining *Wolbachia*-mediated antiviral protection in mosquitoes [29], [30], [32], [35]. On the other hand, chronic immune activation was also proven to be responsible for lifespan reduction in *Drosophila melanogaster* [45].

**Figure 3:**
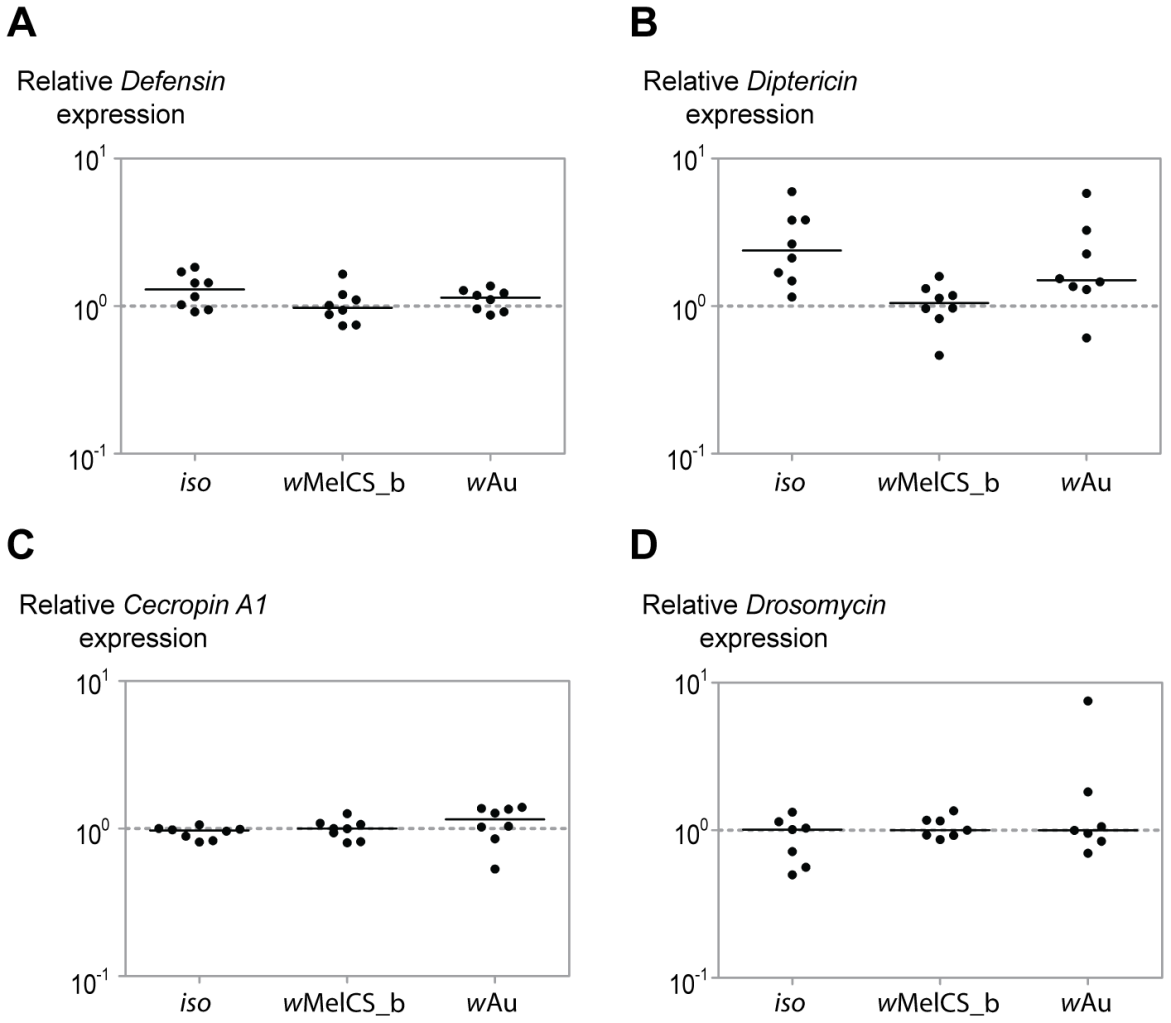
Expression of antimicrobial peptide genes in *Wolbachia*-free *iso*, *w*Au and *w*MelCS_b harbouring flies. qRT-PCR on the RNA collected from 3–6 days-old whole flies performed with the primers specific for *Defensin* (A), *Diptericin* (B) *Cecropin A1* (C) and *Drosomycin* (D). Relative expression of the host antimicrobial peptide genes was calculated using host Rpl32 as a reference. Values are relative to median of samples of *w*MelCS_b. The only statistically significant difference is in *Diptericin* gene expression between *iso* and *w*MelCS_b (pairwise Wilcoxon rank sum test, *p* = 0.006).

To test if chronic immune activation could be responsible for the high antiviral protection and life-shortening by *w*Au we examined the expression of genes encoding antimicrobial peptides (AMPs). We chose AMPs that were previously shown to be highly induced by the presence of exogenous *Wolbachia* [29]–[35], and that represent targets of the two main *Drosophila* immune pathways: Toll and Imd (Figure 3). Quantitative RT-PCR showed that there is no difference between *w*Mel, *w*Au and *iso* in the expression of *Defensin*, *Cecropin A1* and *Drosomycin* (Figure 3). There is also no significant difference between *w*MelCS_b and *w*Au in the expression of *Diptericin*. The lack of an induction of these AMPs by *w*Au indicates that the Toll and Imd pathways are not activated in transinfected *Drosophila melanogaster*. As the expression of the four AMPs is the same in the *w*Au and the *w*MelCS_b infected flies, we could not attribute either the difference in antiviral effect or the lifespan-shortening to the immune activation by exogenous *Wolbachia*. The only statistically significant difference emerging from our analysis was in *Diptericin* gene expression between *iso* and *w*MelCS_b (*p* = 0.006). However, this effect was not observed in the previous studies [31], [36]–[38] and the three other AMPs are not regulated by the presence of wMelCS_b.

Our findings add to previous reports on high AMPs expression not only after *Drosophila* - mosquitoes transfers [29]–[35] but also on *Wolbachia* transferred within the same genus, i.e. *w*AlbB from *A. albopictus* to *A. aegyptii* [33]. The contrast between the effects of these transfers on immunity and lack of immune activation by *w*Au transferred to *D. melanogaster* could be explained in various ways. The first possible explanation may be the phylogenetic distances between the source and target host insect species; the most recent common ancestor of *A. albopictus* and *A. aegyptii* dates to ∼34–42 million years ago [46], while *D. melanogaster* and *D. simulans* diverged only 2.3 million years ago [47]. Therefore, *w*Au could be better pre-adapted to infect *D. melanogaster* inconspicuously. Another explanation is that *D. melanogaster* has co-evolved with *Wolbachia* while *A. aegyptii* natural populations are not infected with this endosymbiont. Thus, *D. melanogaster* may have evolved not to respond to *Wolbachia* infection. This may also explain why *A. albopictus* has a provisional or no immune response to *Wolbachia* somatic transient infection [34]. Finally, *w*Au and *w*Mel might be so similar that the insect's immune system does not perceive *w*Au as foreign.

It would be interesting to know which genetic differences between the closely related *w*Au and *w*MelCS explain the different phenotypes. *w*Au genome is not sequenced, however, several differences between the genome of *w*Au and *w*MelCS are described. *w*Au lacks a 21.86 kb genomic region present in *w*MelCS, named Octomom, which includes genes from WD0506 to WD0518 [15], [21]. This fragment contains genes with domains homologous to eukaryotic proteins (putative *Wolbachia* effector proteins) and many proteins possibly involved in DNA repair and processing. The amplification of this region has been recently proposed to be responsible for the over-replicative phenotype of *w*MelPop *Wolbachia* variant [15], although alternative explanations have been suggested [48]. There are also many other differences in the number or coding sequences of ankyrin repeat genes between *w*Mel strain genomes and *w*Au [21], [49] (see also [15] and [48] for sequence of *w*MelCS). All the above analyses were based on PCR amplification, gene sequencing and DNA hybridization and only the sequencing of the whole *w*Au genome would allow to complete the comparison.

Our study uses *w*Au and one variant of *Wolbachia w*Mel – *w*MelCS_b – in the same *D. melanogaster* genetic background and provides a direct comparison of the protective capabilities of the two strains. We conclude that *w*Au protects better against viral infections – it increases lifespan of virus-infected flies and significantly limits viral replication. Additionally, we have discovered that *w*Au grows exponentially within this host and significantly shortens its lifespan in the absence of viral infection, demonstrating that harbouring this protective endosymbiont is associated with a fitness cost. Testing the expression of selected antimicrobial peptides showed that there is no difference between *w*Mel and *w*Au. Therefore, we could not attribute either the difference in anti-viral effect or the lifespan-shortening to the immune activation by exogenous *Wolbachia*. Our work provides evidence that interspecies *Wolbachia* transfer is not always associated with general immune up-regulation in the recipient host.

## Materials and Methods

The data for iso and *w*MelCS_b in the Figures 1D, 1F, 2C are already published in Chrostek *et al*. 2013. All the remaining data, all statistical analysis and all conclusions are original.

### Fly strains and husbandry

*D. melanogaster* with *w*MelCS_b DrosDel *w* ^1118^ *isogenic* flies and the matching controls without *Wolbachia* were described before [4], [39]. *D. melanogaster* with *w*Au from *D. simulans* Coffes Harbour (CO) was described before [28]. The 1^st^ and 3^rd^ chromosome of the *D. melanogaster* stock with *w*Au were replaced with DrosDel *w* ^1118^ *isogenic* chromosomes using a first and third double balancer line. Next, a second chromosome balancer line was used to replace the 2^nd^ chromosome. As both *Wolbachia* and mitochondria are maternally transmitted the *w*Au, *w*MelCS_b and *Wolbachia*-free iso control lines may have different mitochondria, despite having the same nuclear genetic background. Cleaning the stocks of possible chronic viral infection and gut flora homogenization were performed as in [4], [15]. *Drosophila* were maintained at a constant temperature of 25°C on standard cornmeal diet. All the experiments were performed on 3–6 days-old male flies.

### Long-term survival analysis

The lifespan of different fly lines was tested at 25°C, with 10 flies per vial, and analysed using Cox hazard models as previously reported [15] with the coxme package in R [50]. We considered genotype and repeat of the experiment fixed and replicate vials within the same experiment random.

### Virus production and infection

Viruses were produced, titrated and used to infect flies as before [4], [15]. Infections were performed on 3–6 days-old flies. After the infections 10 flies per vial were kept on food without live yeast at 18°C for DCV or at 25°C for FHV. Survival was monitored daily and vials were changed every 5 days. Statistical analysis was performed the same way as for long-term survival data.

### Nucleic acids extractions and real-time qPCR

DNA for the quantification of *Wolbachia* was extracted using standard phenol-chlorophorm protocol. RNA for assessment of viral titres and gene expression was extracted using Trizol (Invitrogen) with an additional DNAse treatment (Promega) of the AMPs RNA samples prior to cDNA synthesis. cDNA was prepared as described previously [15]. Real-time qPCR reactions were carried out in 7900HT Fast Real-Time PCR System (Applied Biosystems) with the iQ SYBR Green supermix (Bio Rad). Each plate contained three technical replicates of every sample for each set of primers. Primers for *Wolbachia*, DCV and FHV were previously described [15], while primers for AMPs are listed in Table 1. For the four antimicrobial peptides the thermal cycling protocol used was: 50°C for 2 min, 10 min at 95°C and 40 cycles of: 95°C for 30 sec, 59°C for 1 min and 72°C for 30 sec. This was followed by the generation of dissociation curve to verify the specificity of the reactions. Data was analysed in R [50] using Wilcoxon rank sum test with Holm correction for FHV levels, DCV levels at each time point and AMPs levels. The increase of *Wolbachia* variants titre over time was analysed using a linear model (lm) in R [50].

**Table 1:** Primers used to detect AMPs in real-time quantitative PCR experiments.

Target	Forward primer sequence (5′-3′)	Reverse primer sequence (5′-3′)
*Defensin*	TATCGCTTTTGCTCTGCTTG	TGTGGTTCCAGTTCCACTTG
*Diptericin*	ACCGCAGTACCCACTCAATC	CCATATGGTCCTCCCAAGTG
*Cecropin A1*	CATCAGTCGCTCAGACCTCAC	TTCTTCAGCCACCCAGCTTC
*Drosomycin*	taccaagctccgtgagaacc	cagggacccttgtatcttcc

## Supporting Information

Figure S1 **Statistical analysis of survival curves.** (A,B,E) Hazard ratios between either *iso Wolbachia*-free control or *w*MelCS_b carrying line and *w*Au line for: (A) DCV infection, (B) FHV infection, (E) uninfected flies. (C,D,F) Hazard ratios between either *iso* or *w*MelCS_b tetracycline-treated line and *w*Au tetracycline-treated line for: (C) DCV infection, (D) FHV infection, (F) uninfected flies. In all panels error bars represent standard errors of the estimated hazard ratios. The only non-significant differences in Cox hazard ratios are: *iso* tet vs. *w*Au tet for DCV infection (C) and both *iso* tet and *w*MelCS_b tet vs. *w*Au tet for FHV infection (D).
(TIF)
