# Supplementary figures and images for: High Anti-Viral Protection without Immune Upregulation after Interspecies Wolbachia Transfer
Source: PLoS One. 2014 Jun 9;9(6):e99025. doi: 10.1371/journal.pone.0099025 (PMC4049622; doi:10.1371/journal.pone.0099025)

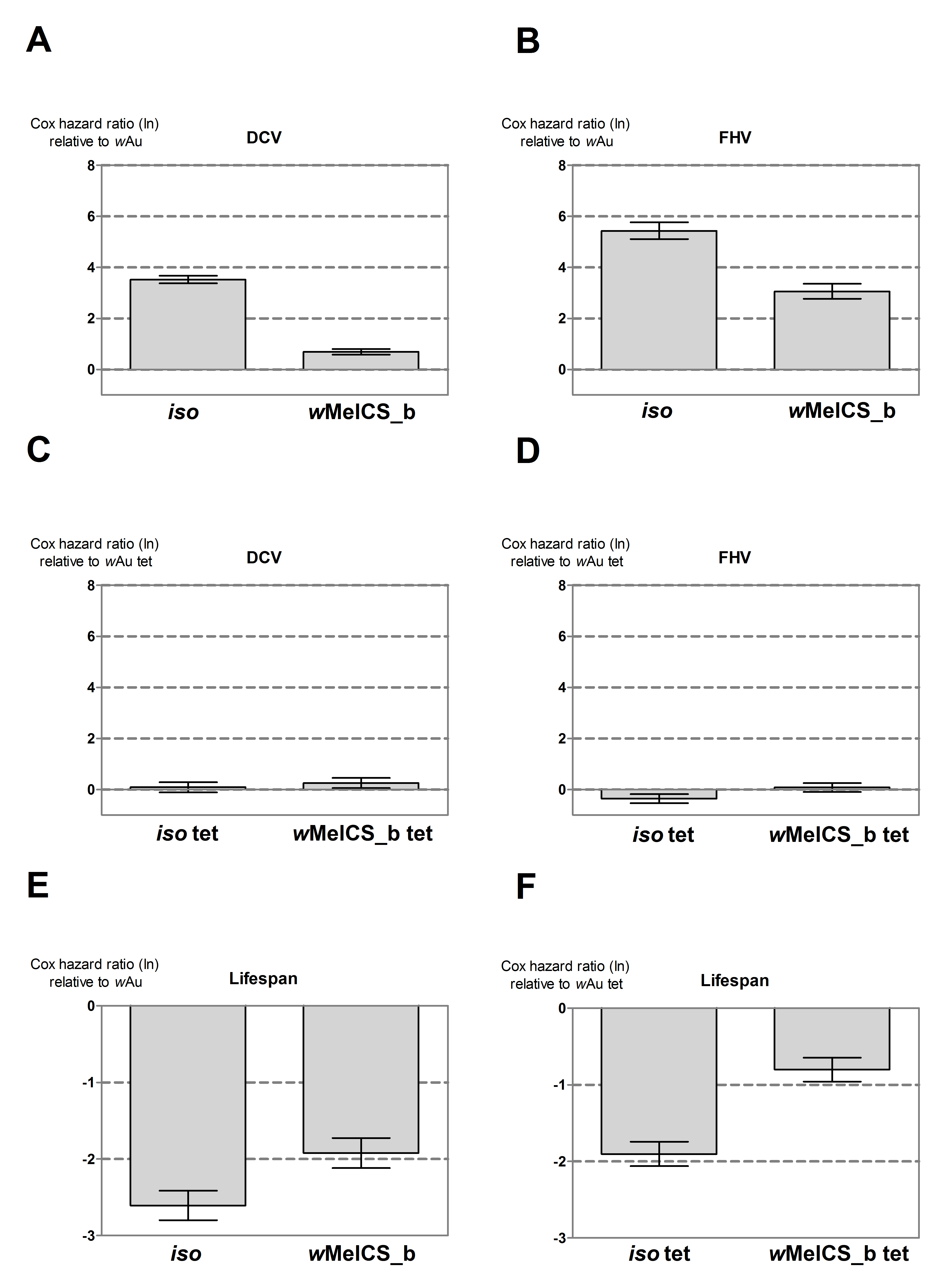

Supplement: Figure S1 — Statistical analysis of survival curves. (A,B,E) Hazard ratios between either iso Wolbachia-free control or wMelCS_b carrying line and wAu line for: (A) DCV infection, (B) FHV infection, (E) uninfected flies. (C,D,F) Hazard ratios between either iso or wMelCS_b tetracycline-treated line and wAu tetracycline-treated line for: (C) DCV infection, (D) FHV infection, (F) uninfected flies. In all panels error bars represent standard errors of the estimated hazard ratios. The only non-significant differences in Cox hazard ratios are: iso tet vs. wAu tet for DCV infection (C) and both iso tet and wMelCS_b tet vs. wAu tet for FHV infection (D). (TIF) [file pone.0099025.s001.tif]
